# Accelerated hematopoietic mitotic aging measured by DNA methylation, blood cell lineage, and Parkinson’s disease

**DOI:** 10.1186/s12864-021-08009-y

**Published:** 2021-09-26

**Authors:** Kimberly C Paul, Alexandra M Binder, Steve Horvath, Cynthia Kusters, Qi Yan, Irish Del Rosario, Yu Yu, Jeff Bronstein, Beate Ritz

**Affiliations:** 1 Department of Neurology, David Geffen School of Medicine at UCLA, Los Angeles, California USA; 2 Population Sciences in the Pacific Program, University of Hawaii Cancer Center, Honolulu, Hawaii USA; 3 Department of Epidemiology, UCLA Fielding School of Public Health, Los Angeles, California USA; 4 Department of Human Genetics, David Geffen School of Medicine at UCLA, Los Angeles, California USA

**Keywords:** Parkinson’s Disease, Mitotic Age, Epigenetics, DNA methylation, Progression

## Abstract

**Background:**

Aging and inflammation are important components of Parkinson’s disease (PD) pathogenesis and both are associated with changes in hematopoiesis and blood cell composition. DNA methylation (DNAm) presents a mechanism to investigate inflammation, aging, and hematopoiesis in PD, using epigenetic mitotic aging and aging clocks. Here, we aimed to define the influence of blood cell lineage on epigenetic mitotic age and then investigate mitotic age acceleration with PD, while considering epigenetic age acceleration biomarkers.

**Results:**

We estimated epigenetic mitotic age using the “epiTOC” epigenetic mitotic clock in 10 different blood cell populations and in a population-based study of PD with whole-blood. Within subject analysis of the flow-sorted purified blood cell types DNAm showed a clear separation of epigenetic mitotic age by cell lineage, with the mitotic age significantly lower in myeloid versus lymphoid cells (p = 2.1e-11). PD status was strongly associated with accelerated epigenetic mitotic aging (AccelEpiTOC) after controlling for cell composition (OR = 2.11, 95 % CI = 1.56, 2.86, p = 1.6e-6). AccelEpiTOC was also positively correlated with extrinsic epigenetic age acceleration, a DNAm aging biomarker related to immune system aging (with cell composition adjustment: R = 0.27, *p* = 6.5e-14), and both were independently associated with PD. Among PD patients, AccelEpiTOC measured at baseline was also associated with longitudinal motor and cognitive symptom decline.

**Conclusions:**

The current study presents a first look at epigenetic mitotic aging in PD and our findings suggest accelerated hematopoietic cell mitosis, possibly reflecting immune pathway imbalances, in early PD that may also be related to motor and cognitive progression.

**Supplementary Information:**

The online version contains supplementary material available at 10.1186/s12864-021-08009-y.

## Introduction

The human blood-system performs numerous vital functions, including the circulation of oxygen and nutrients, temperature homeostasis, and constant immune surveillance of the entire body [1]. As a result, blood cells must be in constant supply and hundreds of billions of cells are made daily to maintain normal function [2]. Meeting the demand for renewal falls on a relatively small pool of hematopoietic stem cells (HSC) that give rise to all hematopoietic and immune cells through a process of organized, stepwise lineage commitment [3]. To maintain steady-state hematopoiesis (formation and development of blood cells), the HSCs are mostly quiescent, while a series of progenitor cells actively proliferate to contribute the bulk of expansion in cell numbers on a daily basis [3–5]. A number of factors can directly influence this process, including inflammation, which is now recognized as an important regulator of HSC biology and hematopoiesis [6, 7]. While short-term, acute hematopoietic responses to pro-inflammatory signals are critical for dealing with a range of inflammatory insults (i.e. infection, tissue damage, etc.), long-term, chronic pro-inflammatory states can age the hematopoietic system, leading to functional decline in both the innate and adaptive immune systems and a skew towards the myeloid-lineage in output [3, 7].

Immune dysregulation and inflammation along with aging are important components of Parkinson’s disease (PD) pathogenesis [8]. Pathologically, PD is characterized by the progressive death of dopaminergic neurons in the *substantia nigra* and the presence of Lewy bodies, intraneuronal aggregates composed of misfolded α-synuclein (αSyn) [9, 10]. There is now ample research that shows that important, systemic immune signals originating outside the brain contribute to PD pathogenesis [11, 12]. Immunosenescence, defined as age-related changes in the immune system, and inflamm-aging, or chronic, low-level inflammatory states, have also been widely linked to neurodegenerative changes and PD, summarized in a number of meta-analyses and reviews [13–18]. Recent reports further indicate that inflamm-aging propagates from the periphery to the brain and vice versa [19]. Additionally, αSyn is notably also widely expressed and abundant in hematopoietic cells as well as neurons [20]. While our understanding of the function of αSyn both peripherally and within the central nervous system (CNS) is still developing, several studies have indicated that it may play an important role in the hematopoietic system related to exo- and endocytosis, apoptosis, autophagy, maturation, and differentiation of hematopoietic cells [20–23]. Thus, there is good rationale to study the intersection between inflammation, aging, and hematopoiesis in PD.

Changes in DNA methylation (DNAm) patterns have been observed to track cell divisions and reflect the proliferative history of different tissues [24–26]. During cell division, DNAm changes occur that appear to accumulate in the stem cells of a tissue in line with and representing the stem cell division rate and chronologic age [24, 27]. These DNAm changes are the basis for the DNAm epigenetic mitotic clock, “epiTOC” (Epigenetic Timer of Cancer), a biomarker that uses methylation patterns to provide an estimate of the relative stem cell division rate of a tissue in an individual [24]. This epigenetic mitotic clock enumerates cellular proliferation of the tissue (i.e. the number of cell divisions) and records the acceleration of the mitotic “tick rate”, or measure of cell divisions, beyond what would be expected with aging based on controls [24]. The epiTOC epigenetic mitotic tick rate has been found to be universally accelerated in cancer tissues and pre-cancerous lesions [24]. Here we propose that the epigenetic mitotic tick rate, tracking the mitotic history of circulating leukocytes with whole-blood DNAm, may also have intriguing implications for Parkinson’s and other diseases of aging with inflamm-aging and immune-related components.

Furthermore, while the epigenetic mitotic clock represents the history of cell divisions of blood cells, epigenetic aging clocks (i.e. DNAm biomarkers of aging), are reflective of the biologic aging process of the tissue [28]. Our previous research indicates that PD patients show more advanced biologic aging markers (i.e. faster biologic than chronologic aging) than controls, with accelerated immune system aging showing the strongest associations of the blood-based measures [29–31]. Alterations in immune profiles in PD patients measured with blood epigenetics have since been replicated [32]. We will now further investigate the proliferative history of blood through the epigenetic mitotic clock, and assess how this relates to immune system aging in relation to PD.

## Background and Aims

**Fig. 1:**
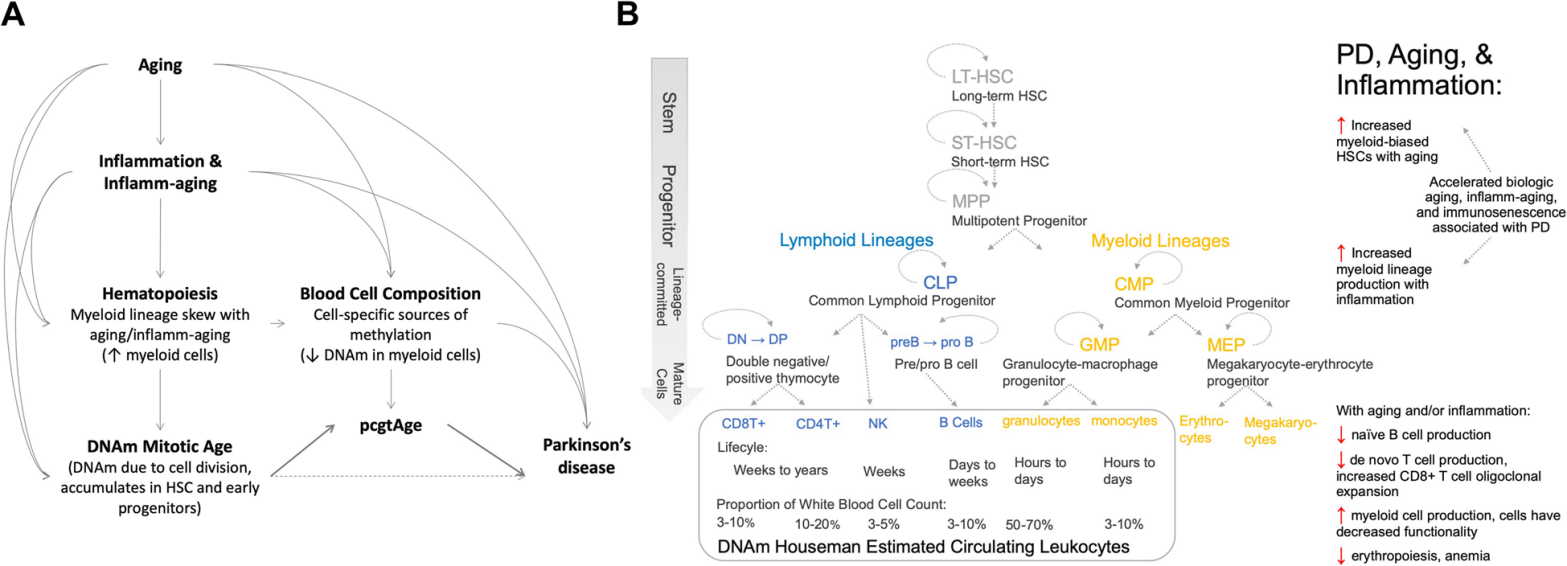
**(A)** Conceptual model of the relationship between mitotic age and the estimated epigenetic mitotic age (epiTOC *pcgtAge*), aging, inflammation/inflamm-aging, hematopoiesis, and Parkinson’s disease. **(B)** Current understanding of hematopoietic cell lineages for the circulating leukocytes estimated with the Houseman method. Cell lifespan and the normal proportion of cells in white blood cell (WBC) counts are displayed in the grey box, as well as the suggested influence of aging and inflammation. See A. Wickrema 2009 for more detail[4]

We hypothesize that PD patients exhibit an accelerated hematopoietic mitotic tick rate relative to controls independent of age, which is reflective of chronic, low-level systemic immune activation, inflammation, and PD-specific pathogenesis. Accordingly, we aimed to investigate whether the epigenetic mitotic tick rate, as an indicator of the proliferative history of blood and hematopoiesis, is associated with early PD and longitudinal PD symptom development among patients. Our analysis and conceptual model can be viewed in Fig. 1A.

Several key concepts are directly relevant to this analysis (Fig. 1B): (1) Aging and inflammation are both associated with a myeloid-bias in hematopoiesis leading to increased numbers of progenitor and mature myeloid cells (aging is associated with myeloid-biased HSCs and inflammation enhances myeloid-lineage production) [3]. (2) The hematopoietic expansion of cell populations is achieved mainly through vast, daily proliferation of progenitor cells at different stages of development, while adult HSCs replicate relatively slowly (estimated every 40 weeks; mostly quiescent), to minimize accumulation of mutations in these parent cells [33, 34]. Given differences in cell turnover rate, the number of cells required daily for homeostasis, and lineage-dependent daily proliferation, different cell types within a whole-blood sample likely have different proliferative histories. (3) DNAm heterogeneity exists between blood cell types and cellular composition may explain substantial variability observed in whole-blood DNAm [35].

Thus, our aims are two-fold, first, define the influence of blood cell lineage and composition on the epiTOC estimated mitotic age using DNAm from purified blood cells, and then, with whole-blood DNAm, associate epigenetic mitotic aging with PD considering the impact of cell composition.

## Results

For analysis we used two data sources, DNAm from flow-sorted purified blood cell types [36] and data from the Parkinson’s disease, Environment, and Genes (PEG) study [37], a population-based study of PD (n = 807) [37]. Characteristics of the PEG study analysis population can be found in Table 1. Of the 807 participants included in the study (n = 569 PD patients and n = 238 population-controls), 62.6 % of patients and 53.4 % of controls were male. The mean age at blood draw was 67.5 years (SD = 12.8) for controls and 70.5 years (SD = 9.8) for PD patients, and the mean PD duration at blood draw (i.e. time from PD diagnosis to blood draw) for the patients was 2.7 years (SD = 2.0).

**Table 1:** Study population characteristics: PEG participants with DNA methylation data (*n* = 807)

	PD patients (*n* = 569)	Controls (*n* = 238)
	Mean (SD) or n (%)	
Age at Blood Draw (SD)	70.5 (9.8)	67.5 (12.8)
Male Sex (%)	356 (62.6)	127 (53.4)
Ancestry (%):		
White	468 (82.4)	228 (95.8)
Hispanic	84 (14.8)	9 (3.8)
Never Smoker (%)	301 (53.2)	96 (40.3)
Ex-Smoker (%)	240 (42.4)	125 (52.5)
Current Smoker (%)	25 (4.4)	17 (7.1)

We calculated the DNAm-based mitotic age of blood, denoted by *pcgtAge*, using the epiTOC model based on published methods [24]. We regressed *pcgtAge* on chronologic age to remove the variation explained by age, using a linear regression model, defining AccelEpiTOC as the corresponding raw residual.

### DNAm Epigenetic mitotic age differs by myeloid and lymphoid lineage

Whole blood consists of many distinct cell populations with varying proportions, important differences in DNAm across cell types, and different rates of cell turnover and proliferation (Fig. 1B) [36]. In order to assess the influence of cell heterogeneity on the epiTOC estimated the mitotic age (*pcgtAge*), we used paired, Illumina 450k DNAm data from 10 different cell populations in blood, from six, adult male donors (mean age 38 ± 13.6 years), including DNAm from flow-sorted myeloid cells (granulocytes, neutrophils, eosinophils, and CD14 + monocytes) and lymphocytes (CD8 + and CD4 + T cells, CD56 + natural killer cells, and CD19 + B cells); GEO accession number GSE35069 [36]. We then calculated the mitotic age (*pcgtAge*) based on DNAm from the different cell types within the same individual.

**Fig. 2:**
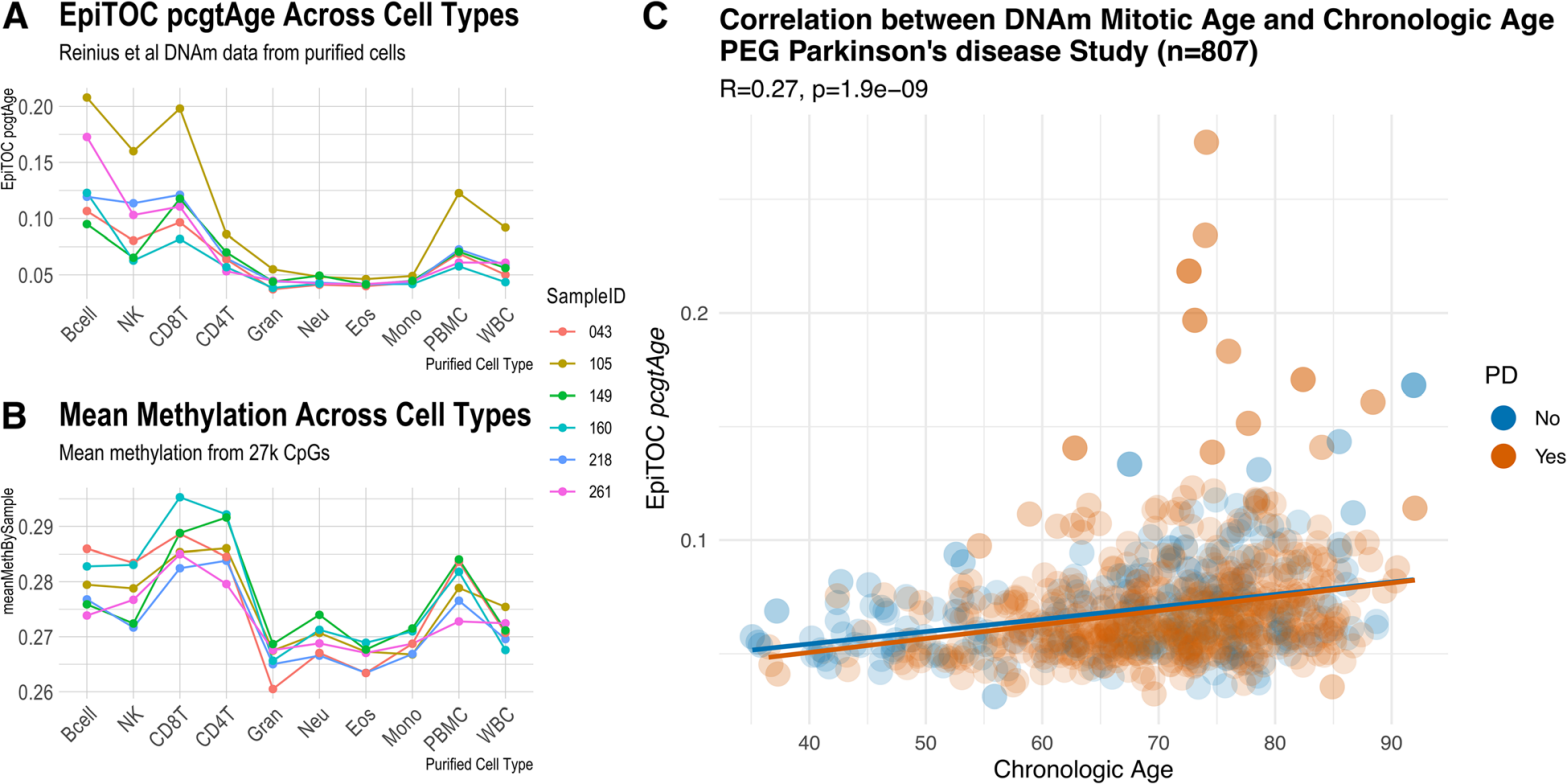
EpiTOC and Blood Cell Composition.** (A)** EpiTOC *pcgtAge* based on DNAm from 10 purified cell types, using n = 6 subjects with paired data. Line graph displays the calculated epiTOC *pcgtAge* at each cell types, with each line representing one participant. **B)** Mean methylation level across 27k CpGs (Illumina 27k array) based on DNAm from the 10 purified cell types. **C)** Correlation between epiTOC *pcgtAge* and chronologic age among the PEG study participants (n = 807)

Within subject there was a clear separation in mitotic age by blood cell lineage (Fig. 2 A). *pcgtAge* was significantly lower among the myeloid cell types (granulocytes, eosinophils, neutrophils, and monocytes) versus lymphoid cells (Bcells, CD4 + and CD8 + T cells, and NK cells) within the same individuals (β for myeloid lineage relative to lymphoid= -0.06, SE = 0.007, p = 2.1e-11 (Supplemental Table 1)). That is, within subject, the *pcgtAge* based on DNAm from CD8 + T cells, for example, was considerably higher on average than the *pcgtAge* based on DNAm from granulocytes (paired t-test, mean difference between CD8 + T cells and granulocytes: 0.08 (95 % CI 0.04, 0.11)).

In fact, as expected from the visual comparisons, when modeling *pcgtAge* in a repeated measure mixed effects model (lme R function), with cell type as the predictor and a random effect for subject, all cell types except Bcells were significantly associated with a lower *pcgtAge* relative to CD8 + T cells (Supplemental Table 1). Interestingly, when we assessed the mean methylation of 27k CpGs (Illumina 27k array) across cell types, we observed a similar pattern, where the mean methylation was significantly lower among myeloid cells relative to lymphoid (β=-0.02, SE = 0.001, p = 9.3e-15; Supplemental Table 2; Fig. 2B). This supports a previous observation of DNAm depletion in cells of the myeloid-lineage, perhaps as a mechanism of lineage-commitment [38].

In the PEG study with whole blood DNAm, we estimated the proportion of CD8 + T cells, CD4 + T cells, natural killer, B cells, monocytes, and granulocytes [39] and also counts of exhausted CD8 + T cells (defined as CD28-CD45RA-), naïve CD4 + T and naïve CD8 + T cells (defined as CD45RA + CCR7+), and plasmablasts [40, 41]. We first correlated *pcgtAge* with chronologic age, and observed *pcgtAge* was positively correlated with age (Fig. 2 C; R = 0.27, p = 2.7e-15 (Rsq = 0.07)) to a similar degree as previously reported by Yang el al. (Discovery Rsq = 0.07; Replication Rsq = 0.13 [24]). In stratified analysis, *pcgtAge* was slightly more positively correlated with age among controls than PD patients (control: R = 0.34, p = 5.5e-8; PD: R = 0.25, *p* = 1.9e-9).

**Fig. 3:**
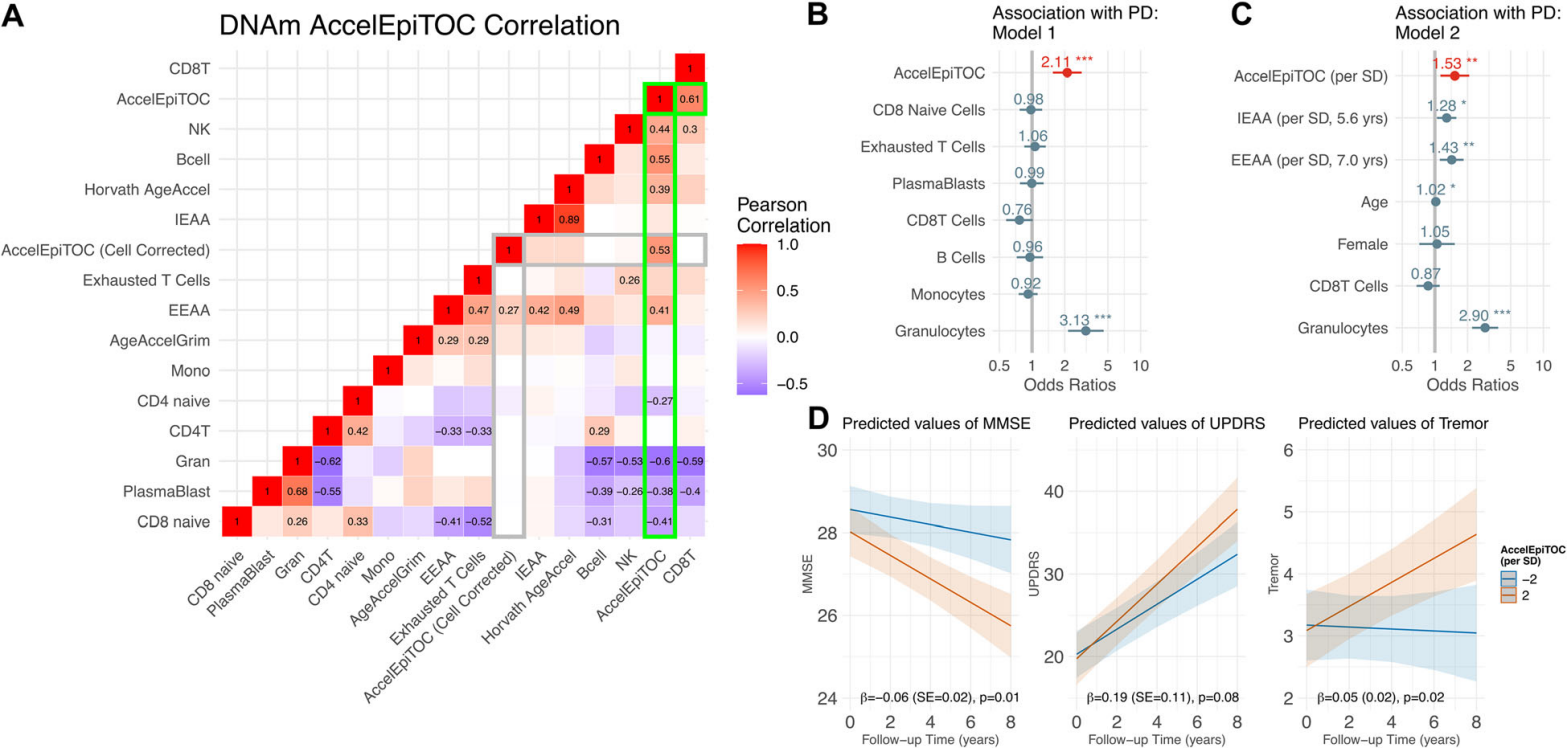
**(A)** Correlations between AccelEpiTOC, DNAm measures of blood cell composition, and other DNAm age acceleration measures in PEG, based on DNAm from whole blood (n = 807). Correlations with |R| ≥ 0.25 included as text **(B & C)** Results from a logistic regression, with AccelEpiTOC predicting PD. **(B)** Model 1: PD association for AccelEpiTOC, adjusting for measures of blood cell composition (all blood cell estimates per SD) and other covariates (below) with. **(C)** Model 2: PD association for AccelEpiTOC and DNAm epigenetic age measures, adjusting for blood cell composition (all blood cell estimates per SD) and other covariates. For both models, all terms are included in the same logistic regression model, also adjusting for age, sex, smoking history, AIMs derived ancestry (European/Hispanic ancestry), and two PCs for DNAm technical variation. The epigenetic mitotic age acceleration (AccelEpiTOC) was centered around zero and scaled per SD. (**D)** Epigenetic mitotic age acceleration and signs of PD symptom progression: predicted change on MMSE, UPDRS-III; and the Tremor UPDRS-III sub-score over follow-up at two levels of AccelEpiTOC (± 2 SD from mean (0)). The y-axis displays the predicted value of the three exam measures, including MMSE (higher score indicates higher cognitive performance), UPDRS-III (higher score indicates more motor symptoms, assessed by neurologist), and UPDRS-III tremor (tremor sub-score of the UPDRS III; higher score indicates more tremor motor symptoms). Analysis based on symptom progression among PD patients only. Results based on linear repeated measures mixed models including an interaction between epigenetic mitotic aging and time to assess how epigenetic mitotic aging influences change on the exams over time. Models control for age, sex, smoking history, race/ethnicity (AIMs derived European/Hispanic ancestry), PEG study wave, PD duration at baseline, measures of blood cell composition (CD8.naive, CD8pCD28nCD45Ran, plasmablast, CD8 + T cells, B cells, monocytes, and granulocytes), and the two PCs for DNAm technical variation. β (SE) term shown: interaction between AccelEpiTOC*time, representing yearly change on predicted exam score according to AccelEpiTOC. Epigenetic mitotic age acceleration (AccelEpiTOC) was centered around zero and scaled per SD

Pairwise Pearson correlations (R) between AccelEpiTOC (i.e. *pcgtAge* after excluding the variation explained by chronologic age) and all DNAm cell composition markers can be visualized in Fig. 3 A. AccelEpiTOC was negatively correlated with the proportion of granulocytes and positively correlated with the proportion of CD8 + T cells, natural killer cells, and Bcell lymphocytes. AccelEpiTOC was also negatively correlated with the counts of plasmablast and CD8 naïve cells. Given the purified cell results (Fig. 2 A & 2B), the correlation directions are as expected. For instance, AccelEpiTOC is strongly negatively correlated with granulocytes (R=-0.60, p = 1.3E-79) in the PEG study. When assessing DNAm in whole-blood in PEG, those individuals with a higher percentage of granulocytes will have their whole blood mean *pcgtAge* weighted more heavily by the granulocyte specific *pcgtAge*, which was substantially lower than the *pcgtAge* from lymphoid cells within-subject (Fig. 2 A).

### Accelerated epigenetic mitotic aging in Parkinson’s disease

PD status was strongly associated with AccelEpiTOC when controlling for blood cell composition (Fig. 3B). Specifically, one standard deviation (SD) increase in AccelEpiTOC was associated with a two-fold increased risk of being a PD patient, indicating the PD patients exhibited a more accelerated mitotic tick rate in blood than controls (AccelEpiTOC OR per SD = 2.11, 95 % CI = 1.56, 2.86, p = 1.6e-6), after controlling for age, sex, ancestry, blood cell composition, and two principal components (PCs) to control for potential technical variation in the DNAm. For reference, there was no association without controlling for blood cell composition (cell-unadjusted AccelEpiTOC OR per SD = 0.93, 95 % CI = 0.80, 1.08, p = 0.35). Due to the strong negative correlation between granulocytes and AccelEpiTOC and the strong positive association between granulocytes and PD, negative bias due to confounding by cell composition is expected [42]. The best fitting model for PD included only granulocytes and CD8 + T cells as sufficient to control for cell type heterogeneity (Supplemental Fig. 1). The results stratified by sex are shown in Supplemental Table 3. The AccelEpiTOC association with PD was observed in both men and women (Men: AccelEpiTOC OR per SD, OR = 2.34, 95 % CI = 1.54, 3.55; Women, OR = 1.86, 95 % CI = 1.17, 2.95).

AccelEpiTOC was also positively correlated with two different measures of epigenetic age acceleration (EAA) in our sample (Fig. 3 A): the pan-tissue epigenetic clock (Horvath AgeAccel) and extrinsic epigenetic age acceleration (EEAA), a measure of biologic aging in immune related components based on the Hannum clock [43] that is somewhat dependent on leukocyte concentrations known to change with age [29]. We have previously shown that PD patients also show faster EEAA and IEAA (intrinsic epigenetic age acceleration, which is the Horvath epigenetic aging measure independent of blood cell counts) [29]. Therefore, we modeled PD including AccelEpiTOC, IEAA, and EEAA along with blood cell composition and other covariates in the same model. The three epigenetic biomarkers were each independently associated with PD (Fig. 3 C): per SD increase in each measure, AccelEpiTOC OR = 1.53 (95 % CI = 1.12, 2.07, p = 0.007), IEAA OR = 1.28 (95 % CI = 1.04, 1.58, p = 0.02), EEAA OR = 1.43 (95 % CI = 1.11, 1.84, p = 0.006). EEAA, which represents accelerated epigenetic aging of immune factors, in particular, was related to AccelEpiTOC (from best fit linear regression model of AccelEpiTOC, EEAA β = 0.27, SE = 0.03, p = 2.2e-25; Supplemental Table 4). The results with the three epigenetic biomarkers stratified by sex are shown in Supplemental Table 5.

To assess progression, we relied on 336 PD patients with repeated examinations of the UPDRS-III and MMSE (2–4 follow-up exams and a mean follow-up of 4.7 years (SD = 2.8)), and used repeated measures linear regression, controlling for age, sex, ancestry, blood cell composition, the two technical variation PCs, PD duration at baseline, PEG patient recruitment wave, education, and baseline exam score. AccelEpiTOC measured at baseline was suggestively associated with longitudinal decline over follow-up on both exams. Specifically, AccelEpiTOC was associated with faster decline on the MMSE (AccelEpiTOC *time (per year) β=-0.06, SE = 0.02, p = 0.01; Fig. 3D) and perhaps faster development of motor symptoms as measured by the UPDRS-III (AccelEpiTOC *time β = 0.19, SE = 0.11, p = 0.08; Fig. 3D), with the UPDRS-III tremor sub-score showing the strongest association among motor symptom domains (β = 0.05, SE = 0.02, *p* = 0.02; Fig. 3D).

## Discussion

In the current study, our findings suggest that PD patients exhibit a considerably accelerated hematopoietic mitotic tick rate (AccelEpiTOC) compared with age-similar community controls, as measured by the epiTOC mitotic clock. This clock estimates the relative stem cell division rate of a tissue, blood in the present study, based on DNAm [24]. This association was apparent after we removed the variation in epiTOC *pcgtAge* explained by age and corrected for blood cell composition. In fact, our findings in purified cells indicate that epigenetic mitotic age is strongly dependent on blood cell lineage, which can thus act as a strong confounder. We also observed associations between accelerated epigenetic mitotic aging and accelerated epigenetic immune system aging (EEAA). This is in line with our hypothesis, that accelerated epigenetic mitotic aging in this elderly PD population is at least partially reflective of more immune activation, as AccelEpiTOC and EEAA likely represent both age-related and disease-related immune changes. Furthermore, among PD patients, AccelEpiTOC was also associated with longitudinal cognitive decline and motor symptom increases as measured by changes on the MMSE and UPDRS-III scores.

HSCs and the different lineages of progenitor cells provide a constant supply of blood cells, which, depending on the cell type, turn over relatively quickly (hours to days; e.g. granulocytes) to more slowly (months to even years; e.g. memory T cells and B cells) [44]. In healthy adults, leukocyte populations are generally maintained at a reasonably constant size through steady-state hematopoiesis, reflecting a balance between blood cell production and loss [4, 45]. The importance of maintaining normal hematopoiesis in aging and age-related diseases is increasingly being recognized [46]. Inflammation exerts a strong influence on hematopoiesis. All blood cell types contribute to the initiation and resolution of inflammatory events [6], and these responses are often accompanied by systemic changes in blood cell composition, including overproduction of myeloid cells as “first-responders” (e.g. granulocytes, monocytes) [6, 47]. Pro-inflammatory cytokines, including interleukin (IL)-1, tumor necrosis factor-α (TNF-α), interferons (IFNs), and others, can upregulate or suppress the normal hematopoietic output [6, 48]. Over time, as the DNAm changes that arise during cell division accumulate throughout the lineage of HSCs and progenitor populations (e.g. multipotent progenitors and common myeloid or lymphocyte progenitors), alterations in the hematopoietic output due to sustained, low-level inflammation, as hypothesized in PD, will be reflected in the mitotic tick rate of the blood tissue accordingly.

In PD, hematopoiesis or alterations in normal blood turnover rates have not been widely studied, most certainly due to the difficulty of quantifying stem cells and progenitors and their turnover for epidemiologic studies. However, one study of 123 individuals investigated this by establishing a colony-forming cell assay and compared HSCs extracted from venous blood of Parkinson’s patients and controls [49]. The authors reported a strong upregulation in the percentage of monocyte precursors and early granulocyte/monocyte precursors in the peripheral blood of PD patients [49]. Our methylation-based results also support that PD patients have a different leukocyte profile from controls, most notably they exhibit a higher proportion of granulocytes and lower proportion of lymphocytes [29]. Here we show for the first time that the DNAm estimated hematopoietic mitotic tick rate itself is actually faster in patients than controls. That is, the PD patients not only differ in the proportions of blood cell types from controls, but their hematopoietic cells have indeed been proliferating more on average, based on the epiTOC *pcgtAge* mitotic tick rate.

Neuroinflammation has been widely and consistently connected to neurodegeneration, with several lines of evidence also pointing toward peripheral immune system involvement. A meta-analysis of measured, peripheral inflammatory cytokine levels across 25 studies, found higher levels of IL-6, IL-1β, IL-2, IL-10, TNF, and C‐reactive protein in PD patients relative to controls [16]. PD patients may also have altered phagocytic activity in peripheral monocytes [50, 51]. A recent cell line study showed that peripheral blood derived immune cells from PD patients survived for a shorter time in culture, and patients’ cells were less responsive to stimulation than those of controls [17]. Furthermore, studies have also shown that neurodegenerative disease patients experience lymphocyte infiltration from the periphery in the central nervous system (CNS) [15, 52], challenging the conventional belief that the CNS is “immune privileged”, i.e. protected from peripheral immune mediators. It is unclear whether αSyn may be involved in the hematopoietic alterations in PD apart from inducing inflammation. However, αSyn is expressed in erythrocytes, T and B lymphocytes, monocytes, natural killer (NK) cells, and megakaryocytes [53], and knock-out mice have shown a number of hematologic abnormalities [20]. Studies have linked αSyn to the hematopoietic system through exo- and endocytosis, apoptosis, autophagy, maturation, and differentiation of hematopoietic cells [20–23]. For instance, in the absence of αSyn, mice showed hematologic abnormalities including anemia and smaller platelets, reduced B cell lymphopoiesis, and defects in immunoglobulin G production and in the development of T lymphocytes [21].

Thus, while there is biologic plausibility to support our findings, a brief discussion of the epiTOC model and its limitations is warranted (we also refer the reader to more in-depth discussions [24, 54, 55]). The epiTOC *pcgtAge* score is based on DNAm over 385 promoter polycomb group target (PCGT) CpGs, all initially unmethylated in fetal tissues and which exhibit hypermethylation with aging. Increases in DNAm at these sites define the epiTOC tick rate, which correlates with stem cell division rates across a number of normal tissues. EpiTOC was found to be universally accelerated in cancer and pre-cancer tissues [24]. The model assumes that changes in methylation at the informative loci occurs via mitosis, which would lead to accumulation in the stem cells and progenitors which is passed down the lineage to circulating blood cells. However, other sources of methylation are possible. Additionally, the model does not consider the size of the hematopoietic cell populations (i.e. concentration of the progenitor populations) and how this might influence the tick rate.

Our analysis of purified cells suggests that the epiTOC *pcgtAge* varies quite considerably with blood cell type within the same person, but there is a clear separation between lymphocyte and myeloid cell types. In fact, in parallel, mean methylation across 27k CpGs also varied considerably, and in a similar pattern. This is not entirely unexpected. A previous report of DNA methylation dynamics across 17 hematopoietic cell types found that the distribution of DNA methylation levels was similar across all stem and progenitor cell types, while there was a shift toward lower methylation levels in differentiated cells of the myeloid lineage [38]. This study also reported that DNA methylation levels at regulatory and many other genomics regions were on average lower in myeloid progenitors and differentiated myeloid cells relative to cells of the lymphocyte lineage [38]. It is therefore possible that this broader DNAm depletion is contributing to the lower *pcgtAge* score we observed in myeloid cells. This also supports the notion that it is more predominantly cell composition influencing DNAm levels overall and in turn epiTOC CpGs as well specifically through general DNAm depletion, versus *pcgtAge* influencing cell composition and thus acting as a collider. It is also likely there is some heterogeneity in the mitotic age in different cell types. The vast majority of blood cell expansion is achieved mainly through proliferation of progenitor cells that are at different stages of development. Differences in cell turnover rate, the number of cells required daily for homeostasis, and lineage-dependent daily proliferation, mean different cell types within a whole-blood sample likely have different proliferative histories. This illustrates the need to control for cell heterogeneity in *pcgtAge* analysis using whole blood methylation, which may otherwise exert strong confounding if left unchecked.

## Conclusions

The technology and methodology to enumerate the mitotic history of tissues with DNAm is in its infancy. We expect it will continue to be developed and improve, while addressing existing limitations. The current study provides a first look and presents striking findings implicating accelerated hematopoietic cell mitosis, possibly reflecting imbalances in immune pathways, in early PD that may also contribute to progression. This methylation-based mitotic clock may have utility in assessing immune system contributions to PD onset and progression, and neurodegenerative research in general, allowing us to explore systemic immune contributions to brain disorders and eventually inspire preventative or therapeutic strategies to slow the process.

## Methods

### PD Study Population

PEG is a population-based study of residents of California’s Central Valley, designed first as a case-control study to investigate PD etiology (2001–2007 & 2010–2016; n = 849 PD patients early in disease; n = 1021 population-based controls), and second as a longitudinal cohort with prospective follow-up of PD patients for progression (n = 525, 2–4 follow-up exams and a mean follow-up of 4.7 years (SD = 2.8)) [56, 57]. PD case-control analysis was restricted to n = 807 participants (n = 228 controls and n = 569 PD patients) for whom we currently have blood-based DNAm data available. All patients in PEG were seen by movement disorder specialists (lead by J.B.) at least once at baseline, many on multiple occasions, and confirmed as having probable idiopathic PD based on published criteria [58]. We also have follow-up data for n = 336 of PD patients with methylation data available. PD patient demographic characteristics with/out methylation data were similar: 62 % vs. 65 % male, 77 % vs. 76 % European ancestry, mean age 70.4 years (SD = 11.7) vs. 70.5 years (SD = 9.8). Further information on the study population is provided in the supplemental materials.

DNA was extracted from peripheral whole blood and we profiled and processed DNA samples using the Illumina Infinium 450k platform (486k CpGs) by applying standard settings. DNA methylation β values were preprocessed using the background normalization method from the Genome Studio software and corrected for type I/type II probe bias with BMIQ using the champ.norm function in the ChAMP R package [59]. More detail has been published [60, 61], and the data is available on Gene Expression Omnibus (GEO), accession numbers GSE72774 and GSE72776. To account for possible technical factors due to array or batch effects, we calculated principal components based on the DNAm levels of the 848 control probes included on the 450 K DNAm array. We used the first two PCs, which explained the majority of variation in the probes, to capture technical variation.

Informed consent was obtained from all subjects and the study protocol was approved by the UCLA institutional review board.

### epiTOC epigenetic mitotic clock

We calculated the DNAm-based mitotic age of blood, representing the cell division history reflected within the DNAm, using the epiTOC model based on published methods [24]. The epigenetic mitotic age estimate from epiTOC is denoted by *pcgtAge*, based on the nomenclature by Yang el al. [24]. EpiTOC is a DNAm-based, age-correlative model which approximates a mitotic clock in both normal and cancer tissue [24]. It focuses on 385 CpG promoter sites that localize to Polycomb group target genes that are unmethylated in 11 different fetal tissue types. Increases in DNA methylation at these sites define the epiTOC tick rate, which correlates with the rate of stem cell division in normal tissues as estimated in stem cell research [62]. EpiTOC was trained using Illumina 450k DNAm data from 656 whole blood samples from healthy individuals spanning an age range of over 80 years, correcting for changes in blood cell composition [43], and was validated in an independent 450k dataset of > 300 healthy controls [24]. We regressed *pcgtAge* on chronologic age to remove the variation explained by age, using a linear regression model and defining AccelEpiTOC as the corresponding raw residual (i.e. the difference between the observed value of epiTOC *pcgtAge* minus its expected value). AccelEpiTOC was transformed into units of standard deviation (SD).

### Blood Cell Composition and DNAm from Purified Cells

In order to assess the influence of cell heterogeneity on the epiTOC estimated *pcgtAge*, we calculated *pcgtAge* in paired, Illumina HumanMethylation450 BeadChip data from 10 different cell populations in blood, from six, adult male donors, including DNAm from flow-sorted myeloid cells (granulocytes, neutrophils, eosinophils, and CD14 + monocytes) and lymphocytes (CD8 + and CD4 + T cells, CD56 + natural killer cells, and CD19 + B cells); GEO accession number GSE35069 [36].

In PEG, we estimated whole blood cell (WBC) composition using two different methods. First, we used the Houseman estimation method [39] that estimates the proportion of CD8 + T cells, CD4 + T cells, natural killer, B cells, monocytes, and granulocytes. Second, we employed the Horvath blood cell estimation method [40, 41], to estimate counts of exhausted CD8 + T cells (defined as CD28-CD45RA-), naïve CD4 + T and naïve CD8 + T cells (defined as CD45RA + CCR7+), and plasmablasts. We also estimated DNAm epigenetic age acceleration using three epigenetic aging clocks: the Horvath clock age acceleration (termed as *Horvath AgeAccel*; pan-tissue epigenetic clock) [41]; intrinsic epigenetic age acceleration (*IEAA*; epigenetic aging measure independent of blood cell counts) [29]; and extrinsic epigenetic age acceleration (*EEAA*; measure of biologic aging in immune related components based on the Hannum clock [43] and somewhat dependent on leukocyte concentrations known to change with age) [29].

### Statistical Analysis

To assess the influence of cell composition on epigenetic mitotic age (*pcgtAge*) within person, we used a repeated-measures linear mixed model, treating repeated measures *pcgtAge* across cell types as the outcome and cell type (categorical) as the predictor, with a random effect for subject (n = 6 subjects with 10 *pcgtAge* estimates from different cell types, 60 observations). We also assessed global methylation differences across cell types with a repeated measures model, using mean methylation across 27k CpGs included in the Illumina 27k methylation array.

To assess associations between the epigenetic mitotic tick rate (AccelEpiTOC) and PD status in the PEG study, we used logistic regression to estimate odds ratios (ORs) and 95 % CIs, controlling for age, sex, ancestry derived from ancestry informative markers (AIMS), blood cell composition, and the two technical variation PCs. We selected the most parsimoniously adjusted and best fitting model for PD based on step-wise variable selection and minimized Akaike information criterion (AIC). We assessed the association between AccelEpiTOC and PD symptom progression measured by the Unified Parkinson’s disease Rating Scale, Part III (UPDRS-III, motor symptoms) and Mini-Mental State Exam (MMSE, cognitive symptoms), with repeated-measures linear mixed models. We included an interaction term between AccelEpiTOC and follow-up time (in years) to estimate the change in symptom score over time according to AccelEpiTOC. The regression coefficient (β) for the interaction term with time represents the estimated difference in annual change in outcome score (UPDRS-III or MMSE) according to AccelEpiTOC. We also present Pearson correlations between epiTOC *pctAge*, AccelEpiTOC, blood cell composition, and the epigenetic clock age acceleration measures. All analyses were done using R software.

## Supplementary Information

**Additional file 1.**

**Additional file 2: Supplemental Table 1.** Output from linear mixed effects repeated measures regression model of epiTOC pcgtAge, among 6 participants with DNAm from purified cell types.

**Additional file 3: Supplemental Table 2.** Output from linear mixed effects repeated measures regression model of 27k CpG mean methylation, among 6 participants with DNAm from purified cell types. 

**Additional file 4: Supplemental Table 3.** Output from logistic regression model of PD, with AccelEpiTOC and all covariates, stratified by Sex. All terms included as covariates in the same model. Model 1 in manuscript, stratified by sex.

**Additional file 5: Supplemental Table 4.** Output from the best fit, linear regression model of AccelEpiTOC. Full model shown, AccelEpiTOC is the outcome and all terms listed are included as covariates in the same model.

**Additional file 6: Supplemental Table 5.** Output from logistic regression model of PD, with AccelEpiTOC and all covariates, stratified by Sex. All terms included as covariates in the same model. Model 2, includes other DNAm age markers.

**Additional file 7: Supplemental Figure 1.** Best fitting logistic model of PD.

## Data Availability

The datasets analyzed during the current study are available in the GEO repository, accession numbers GSE72774 and GSE72776 (PEG), and the purified blood cell DNAm data at GSE35069.

## Abbreviations

αSyn: α-synuclein
AIC: Akaike information criterion
AIMs: ancestry informative markers
CD4T: CD4+ T cells
CD8T: CD8+ T cells
CNS: Central nervous system
DNAm: DNA methylation
EEAA: Extrinsic epigenetic age acceleration
Gran: Granulocytes
HSC: Hematopoietic stem cell
IEAA: Intrinsic epigenetic age acceleration
IL: Interleukin
MMSE: Mini-Mental State Exam
NK: CD56+ natural killer cells
PCGT: promoter polycomb group target
PD: Parkinson’s disease
PEG: Parkinson’s Environment and Genes Study
TNF: Tumor necrosis factor
UPDRS-III: Unified Parkinson’s Disease Rating Scale, part III
