# Supplementary material for: Accelerated hematopoietic mitotic aging measured by DNA methylation, blood cell lineage, and Parkinson’s disease
Source: BMC Genomics. 2021 Sep 26;22:696. doi: 10.1186/s12864-021-08009-y (PMC8474781; doi:10.1186/s12864-021-08009-y)
Supplement: Supplementary file 1 — Additional file 1. [file 12864_2021_8009_MOESM1_ESM.docx]

**Supplemental Material**

**PEG Study Population Methods**

Patients were first identified through local neurologists, clinics, and community outreach, and starting in 2010 as part of a pilot PD registry in California. Population-based controls from the same communities were randomly sampled from Medicare lists (before HIPAA enactment) and throughout via residential tax assessor's records. All patients in PEG were seen by movement disorder specialists (lead by J.B.) at least once at baseline, many on multiple occasions, and confirmed as having probable idiopathic PD based on published criteria(58). The patients at baseline were generally early in their disease course (71% diagnosed within 3 years of recruitment; mean PD duration at baseline: 3.0 years (SD=2.6), IQR=1 to 4 years duration) and during follow-up progressed in disease (mean PD duration at latest follow-up of 7.4 years (SD=3.0), IQR=5 to 9 years). Of those not examined during follow-up (n=308), 174 were deceased (50%), 42 refused or could not be re-contacted (12%), and 92 are pending examinations (38%).

Trained interviewers recorded information on demographics, medical history, and lifestyle for all participants. Physical examinations for PD progression were performed by UCLA movement disorder specialists and included motor symptoms documented with the Unified Parkinson’s Disease Rating Scale (UPDRS) part III (higher scores indicate worse motor symptoms), which can be split into sub-scores for bradykinesia, posture/gait, tremor, rigidity and axial symptoms. Cognitive function was assessed with the Mini-Mental State Examination (MMSE; range, 0-30, with lower scores indicating worse cognitive function)(63) at the same neurologic examination.
