# Supplementary material for: Accelerated hematopoietic mitotic aging measured by DNA methylation, blood cell lineage, and Parkinson’s disease
Source: BMC Genomics. 2021 Sep 26;22:696. doi: 10.1186/s12864-021-08009-y (PMC8474781; doi:10.1186/s12864-021-08009-y)
Supplement: Supplementary file 2 — Additional file 2: Supplemental Table 1. Output from linear mixed effects repeated measures regression model of epiTOC pcgtAge, among 6 participants with DNAm from purified cell types. [file 12864_2021_8009_MOESM2_ESM.docx]

| **Supplemental Table 1.** Output from linear mixed effects repeated measures regression model of epiTOC *pcgtAge*, among 6 participants with DNAm from purified cell types. | | | | | | | | | | |
| --- | --- | --- | --- | --- | --- | --- | --- | --- | --- | --- |
|  |  | **Model 1: Lineage as predictor** | | | **Model 1: Cell Type as predictor, REF CD8T Cells** | | | **Model 2: Cell Type as predictor, REF WBC Cells** | | |
| **Cell Types** | **Lineage** | **beta** | **SE** | **p-value** | **beta** | **SE** | **p-value** | **beta** | **SE** | **p-value** |
| **CD8T** | **Lymphoid** | **REF** | | | **REF** | | | 0.061 | 0.010 | 6.31E-07 |
| **Bcell** |  |  |  |  | 0.016 | 0.010 | 1.24E-01 | 0.077 | 0.010 | 2.96E-09 |
| **NK** |  |  |  |  | -0.023 | 0.010 | 3.04E-02 | 0.037 | 0.010 | 8.92E-04 |
| **CD4T** |  |  |  |  | -0.055 | 0.010 | 3.83E-06 | 0.006 | 0.010 | 5.97E-01 |
| **Gran** | **Myeloid** | -0.062 | 0.007 | 2.10E-11 | -0.077 | 0.010 | 3.09E-09 | -0.016 | 0.010 | 1.27E-01 |
| **Neu** |  |  |  |  | -0.077 | 0.010 | 3.57E-09 | -0.016 | 0.010 | 1.37E-01 |
| **Eos** |  |  |  |  | -0.079 | 0.010 | 1.72E-09 | -0.018 | 0.010 | 9.05E-02 |
| **Mono** |  |  |  |  | -0.077 | 0.010 | 3.60E-09 | -0.016 | 0.010 | 1.38E-01 |
| **PBMC** | **Mix** | -0.038 | 0.009 | 9.95E-05 | -0.045 | 0.010 | 8.29E-05 | 0.015 | 0.010 | 1.49E-01 |
| **WBC** |  |  |  |  | -0.061 | 0.010 | 6.31E-07 | **REF** | | |
| We modeled *pcgtAge* in a repeated measure mixed effects model (lme), with cell type as the predictor and a random effect for subject | | | | | | | | | | |
