# Supplementary material for: Accelerated hematopoietic mitotic aging measured by DNA methylation, blood cell lineage, and Parkinson’s disease
Source: BMC Genomics. 2021 Sep 26;22:696. doi: 10.1186/s12864-021-08009-y (PMC8474781; doi:10.1186/s12864-021-08009-y)
Supplement: Supplementary file 3 — Additional file 3: Supplemental Table 2. Output from linear mixed effects repeated measures regression model of 27k CpG mean methylation, among 6 participants with DNAm from purified cell types. [file 12864_2021_8009_MOESM3_ESM.docx]

| **Supplemental Table 2.** Output from linear mixed effects repeated measures regression model of 27k CpG mean methylation, among 6 participants with DNAm from purified cell types. | | | | | | | | | | |
| --- | --- | --- | --- | --- | --- | --- | --- | --- | --- | --- |
|  |  | **Model 1: Lineage as predictor** | | | **Model 1: Cell Type as predictor, REF CD8T Cells** | | | **Model 2: Cell Type as predictor, REF WBC Cells** | | |
| **Cell Types** | **Lineage** | **beta** | **SE** | **p-value** | **beta** | **SE** | **p-value** | **beta** | **SE** | **p-value** |
| **CD8T** | **Lymphoid** | **REF** | | | **REF** | | | 0.016 | 0.002 | 4.22E-11 |
| **Bcell** |  |  |  |  | -0.008 | 0.002 | 5.73E-05 | 0.008 | 0.002 | 1.29E-04 |
| **NK** |  |  |  |  | -0.010 | 0.002 | 4.71E-06 | 0.007 | 0.002 | 1.29E-03 |
| **CD4T** |  |  |  |  | -0.001 | 0.002 | 5.01E-01 | 0.015 | 0.002 | 4.03E-10 |
| **Gran** | **Myeloid** | -0.015 | 0.001 | 9.13E-15 | -0.022 | 0.002 | 6.62E-15 | -0.005 | 0.002 | 7.46E-03 |
| **Neu** |  |  |  |  | -0.018 | 0.002 | 3.71E-12 | -0.001 | 0.002 | 4.59E-01 |
| **Eos** |  |  |  |  | -0.021 | 0.002 | 1.40E-14 | -0.005 | 0.002 | 1.42E-02 |
| **Mono** |  |  |  |  | -0.019 | 0.002 | 9.90E-13 | -0.002 | 0.002 | 2.51E-01 |
| **PBMC** | **Mix** | -0.007 | 0.002 | 8.78E-05 | -0.008 | 0.002 | 1.32E-04 | 0.008 | 0.002 | 5.57E-05 |
| **WBC** |  |  |  |  | -0.016 | 0.002 | 4.22E-11 | **REF** | | |
| We modeled *pcgtAge* in a repeated measure mixed effects model (lme), with cell type as the predictor and a random effect for subject | | | | | | | | | | |
