# Supplementary material for: Accelerated hematopoietic mitotic aging measured by DNA methylation, blood cell lineage, and Parkinson’s disease
Source: BMC Genomics. 2021 Sep 26;22:696. doi: 10.1186/s12864-021-08009-y (PMC8474781; doi:10.1186/s12864-021-08009-y)
Supplement: Supplementary file 4 — Additional file 4: Supplemental Table 3. Output from logistic regression model of PD, with AccelEpiTOC and all covariates, stratified by Sex. All terms included as covariates in the same model. Model 1 in manuscript, stratified by sex. [file 12864_2021_8009_MOESM4_ESM.docx]

| **Supplemental Table 3.** Output from logistic regression model of PD, with AccelEpiTOC and all covariates, stratified by Sex. All terms included as covariates in the same model. Model 1 in manuscript, stratified by sex. | | | | | | | |
| --- | --- | --- | --- | --- | --- | --- | --- |
|  | **Men** | | |  | **Women** | | |
| **Term** | **OR** | **95% CI** | **p.value** |  | **OR** | **95% CI** | **p.value** |
| AccelEpiTOC (per SD) | 2.34 | 1.54, 3.55 | 6.57E-05 |  | 1.86 | 1.17, 2.95 | 8.40E-03 |
| Age | 0.99 | 0.97, 1.02 | 4.57E-01 |  | 1.04 | 1.02, 1.07 | 1.69E-03 |
| RFvoteCaucasian (per SD) | 0.66 | 0.48, 0.90 | 9.66E-03 |  | 0.92 | 0.68, 1.24 | 5.85E-01 |
| Smoker (per SD) | 0.53 | 0.36, 0.78 | 1.41E-03 |  | 0.62 | 0.38, 0.99 | 4.47E-02 |
| CD8 naïve (per SD) | 1.07 | 0.77, 1.48 | 7.05E-01 |  | 0.94 | 0.65, 1.34 | 7.16E-01 |
| CD8pCD28nCD45RAn (per SD) | 1.14 | 0.86, 1.53 | 3.63E-01 |  | 0.98 | 0.66, 1.44 | 9.11E-01 |
| PlasmaBlast (per SD) | 0.73 | 0.50, 1.05 | 8.55E-02 |  | 1.43 | 0.99, 2.08 | 5.97E-02 |
| CD8T (per SD) | 0.68 | 0.47, 1.00 | 5.11E-02 |  | 0.92 | 0.60, 1.41 | 6.98E-01 |
| Bcell (per SD) | 0.91 | 0.63, 1.31 | 5.98E-01 |  | 1.14 | 0.73, 1.77 | 5.75E-01 |
| Mono (per SD) | 0.97 | 0.74, 1.28 | 8.22E-01 |  | 0.88 | 0.65, 1.20 | 4.25E-01 |
| Gran (per SD) | 3.89 | 2.32, 6.54 | 2.83E-07 |  | 2.75 | 1.55, 4.90 | 5.81E-04 |
| PC 1 (per SD) | 1.11 | 0.87, 1.41 | 4.05E-01 |  | 1.13 | 0.86, 1.49 | 3.65E-01 |
| PC 2 (per SD) | 1.10 | 0.85, 1.42 | 4.86E-01 |  | 1.17 | 0.91, 1.50 | 2.15E-01 |
| PC1 and PC2 are principal components to control for DNAm technical variation. | | | | | | | |
