# Supplementary material for: Accelerated hematopoietic mitotic aging measured by DNA methylation, blood cell lineage, and Parkinson’s disease
Source: BMC Genomics. 2021 Sep 26;22:696. doi: 10.1186/s12864-021-08009-y (PMC8474781; doi:10.1186/s12864-021-08009-y)
Supplement: Supplementary file 5 — Additional file 5: Supplemental Table 4. Output from the best fit, linear regression model of AccelEpiTOC. Full model shown, AccelEpiTOC is the outcome and all terms listed are included as covariates in the same model. [file 12864_2021_8009_MOESM5_ESM.docx]

| **Supplemental Table 4.** Output from the best fit, linear regression model of AccelEpiTOC. Full model shown, AccelEpiTOC is the outcome and all terms listed are included as covariates in the same model. | | | |
| --- | --- | --- | --- |
| **Term** | **estimate** | **std.error** | **p.value** |
| PD (yes) | 0.14 | 0.05 | 3.73E-03 |
| EEAA (per SD) | 0.27 | 0.03 | 2.17E-25 |
| Age (per year) | 0.01 | 0.00 | 1.11E-04 |
| CD8 naïve (per SD) | -0.09 | 0.03 | 1.02E-03 |
| CD8pCD28nCD45RAn (per SD) | -0.06 | 0.03 | 3.10E-02 |
| CD8T (per SD) | 0.51 | 0.03 | 1.02E-60 |
| Mono (per SD) | -0.05 | 0.02 | 3.02E-02 |
| Gran (per SD) | -0.12 | 0.04 | 1.51E-03 |
| Bcell (per SD) | 0.36 | 0.03 | 9.64E-30 |
| PC2 (per SD) | -0.07 | 0.02 | 5.80E-04 |
| Model Adjusted R-squared: 0.7127 | | | |
