# Supplementary material for: Accelerated hematopoietic mitotic aging measured by DNA methylation, blood cell lineage, and Parkinson’s disease
Source: BMC Genomics. 2021 Sep 26;22:696. doi: 10.1186/s12864-021-08009-y (PMC8474781; doi:10.1186/s12864-021-08009-y)
Supplement: Supplementary file 6 — Additional file 6: Supplemental Table 5. Output from logistic regression model of PD, with AccelEpiTOC and all covariates, stratified by Sex. All terms included as covariates in the same model. Model 2, includes other DNAm age markers. [file 12864_2021_8009_MOESM6_ESM.docx]

| **Supplemental Table 5.** Output from logistic regression model of PD, with AccelEpiTOC and all covariates, stratified by Sex. All terms included as covariates in the same model. Model 2, includes other DNAm age markers. | | | | | | | |
| --- | --- | --- | --- | --- | --- | --- | --- |
|  | **Men** | | |  | **Women** | | |
| **Term** | **OR** | **95% CI** | **p.value** |  | **OR** | **95% CI** | **p.value** |
| AccelEpiTOC (per SD) | 1.61 | 1.04, 2.48 | 3.12E-02 |  | 1.40 | 0.89, 2.21 | 1.50E-01 |
| IEAA (per SD) | 1.14 | 0.86, 1.51 | 3.64E-01 |  | 1.47 | 1.08, 2.02 | 1.59E-02 |
| EEAA (per SD) | 1.44 | 1.05, 1.98 | 2.35E-02 |  | 1.44 | 0.94, 2.19 | 9.28E-02 |
| Age | 0.99 | 0.97, 1.02 | 6.02E-01 |  | 1.05 | 1.02, 1.07 | 1.85E-04 |
| RFvoteCaucasian (per SD) | 0.65 | 0.47, 0.90 | 9.52E-03 |  | 0.86 | 0.63, 1.16 | 3.11E-01 |
| Smoker (per SD) | 0.55 | 0.37, 0.83 | 3.71E-03 |  | 0.70 | 0.43, 1.12 | 1.37E-01 |
| CD8T (per SD) | 0.79 | 0.55, 1.14 | 2.08E-01 |  | 0.97 | 0.68, 1.38 | 8.76E-01 |
| Gran (per SD) | 3.03 | 2.10, 4.39 | 4.00E-09 |  | 2.96 | 1.90, 4.62 | 1.62E-06 |
| PC 1 (per SD) | 1.15 | 0.89, 1.48 | 2.76E-01 |  | 1.15 | 0.87, 1.52 | 3.27E-01 |
| PC 2 (per SD) | 1.06 | 0.82, 1.37 | 6.77E-01 |  | 1.17 | 0.91, 1.50 | 2.28E-01 |
| PC1 and PC2 are principal components to control for DNAm technical variation. | | | | | | | |
