# Supplementary material for: Accelerated hematopoietic mitotic aging measured by DNA methylation, blood cell lineage, and Parkinson’s disease
Source: BMC Genomics. 2021 Sep 26;22:696. doi: 10.1186/s12864-021-08009-y (PMC8474781; doi:10.1186/s12864-021-08009-y)
Supplement: Supplementary file 7 — Additional file 7: Supplemental Figure 1. Best fitting logistic model of PD. [file 12864_2021_8009_MOESM7_ESM.docx]

**Supplemental Figure 1.** Best fitting logistic model of PD.
